# Functional and Integrative Analysis of the Proteomic Profile of Radish Root under Pb Exposure

**DOI:** 10.3389/fpls.2016.01871

**Published:** 2016-12-15

**Authors:** Yan Wang, Liang Xu, Mingjia Tang, Haiyan Jiang, Wei Chen, Wei Zhang, Ronghua Wang, Liwang Liu

**Affiliations:** National Key Laboratory of Crop Genetics and Germplasm Enhancement, College of Horticulture, Nanjing Agricultural UniversityNanjing, China

**Keywords:** proteomics, radish, iTRAQ, heavy metal, lead stress

## Abstract

Lead (Pb) is one of the most abundant heavy metal (HM) pollutants, which can penetrate the plant through the root and then enter the food chain causing potential health risks for human beings. Radish is an important root vegetable crop worldwide. To investigate the mechanism underlying plant response to Pb stress in radish, the protein profile changes of radish roots respectively upon Pb(NO_3_)_2_ at 500 mg L^−1^(Pb500) and 1000 mg L^−1^(Pb1000), were comprehensively analyzed using iTRAQ (Isobaric Tag for Relative and Absolute Quantification). A total of 3898 protein species were successfully detected and 2141 were quantified. Among them, a subset of 721 protein species were differentially accumulated upon at least one Pb treatment, and 135 ones showed significantly abundance changes under both two Pb-stressed conditions. Many critical protein species related to protein translation, processing, and degradation, reactive oxygen species (ROS) scavenging, photosynthesis, and respiration and carbon metabolism were successfully identified. Gene Ontology (GO) and pathway enrichment analysis of the 135 differential abundance protein species (DAPS) revealed that the overrepresented GO terms included “cell wall,” “apoplast,” “response to metal ion,” “vacuole,” and “peroxidase activity,” and the critical enriched pathways were involved in “citric acid (TCA) cycle and respiratory electron transport,” “pyruvate metabolism,” “phenylalanine metabolism,” “phenylpropanoid biosynthesis,” and “carbon metabolism.” Furthermore, the integrative analysis of transcriptomic, miRNA, degradome, metabolomics and proteomic data provided a strengthened understanding of radish response to Pb stress at multiple levels. Under Pb stress, many key enzymes (i.e., ATP citrate lyase, Isocitrate dehydrogenase, fumarate hydratase and malate dehydrogenase) involved in the glycolysis and TCA cycle were severely affected, which ultimately cause alteration of some metabolites including glucose, citrate and malate. Meanwhile, a series of other defense responses including ascorbate (ASA)–glutathione (GSH) cycle for ROS scavenging and Pb-defense protein species (glutaredoxin, aldose 1-epimerase malate dehydrogenase and thioredoxin), were triggered to cope with Pb-induced injuries. These results would be helpful for further dissecting molecular mechanism underlying plant response to HM stresses, and facilitate effective management of HM contamination in vegetable crops by genetic manipulation.

## Introduction

Heavy metal (HM) contamination from natural, agricultural, and industrial sources has become a worldwide public health concern, which could seriously deteriorate the environment and cause adverse impacts on human health through the food chain (Pourrut et al., 2013; Singh et al., 2016). Lead (Pb) is one of the most abundant HM pollutants with no physiological function, mostly penetrating the plant through the roots and accumulating in different parts (Gupta et al., 2013; Pourrut et al., 2013). Pb can influence various morphological, physiological and biochemical processes leading to a restriction of plant growth, inducing membrane cell damages and, ultimately, to cell death (Sharma and Dubey, 2005; Gupta et al., 2011). Accordingly, plants also have developed diverse defense mechanisms to prevent the toxic effect of heavy metals including generating signal sensing and transduction proteins, compartmenting into the vacuoles and induction of higher levels of metal chelates like a protein complex, organic and inorganic complexes (Gupta et al., 2011, 2013; Thapa et al., 2012).

Radish (*Raphanus sativus* L.), a major member of the Brassicaceae family, is an important annual or biennial root vegetable crop worldwide (Wang and He, 2005). Because the root is considered as the vulnerable part which is easily affected by HM (Wang et al., 2015a), it has become of vital importance to investigate the HM-response mechanisms and explore the molecular regulatory network of tolerance and homeostasis in radish. The identification of the HM-responsive genes or proteins is a fundamental step in understanding the molecular mechanism underlying response to HM stress (Ahsan et al., 2009). In our previous study, NGS-based transcriptome, miRNA and degradome analysis were employed to investigate the expression patterns of genes and miRNAs in radish exposure to Pb stress. A lot of Pb-responsive transcripts, miRNA and its targets were detected, which were predominately involved in stress-related signal sensing and transduction, specific metal uptake and homeostasis, glutathione metabolism-related processes and carbohydrate metabolism-related pathways (Wang et al., 2013, 2015b).

Although transcriptomics provides a useful tool for unraveling gene expression networks at the mRNA level which enhanced our understanding the response of radish under Pb stress, proteomics can offer a new platform for investigating complex biological functions involving large numbers of proteins and provide further insight into posttranscriptional modifications thereby complementing genomics analysis (Ralhan et al., 2008; Ahsan et al., 2009; Wang et al., 2014). In the past two decades, classical two-dimensional electrophoresis (2-DE) technology has been widely employed for protein identification and analysis. However, there were some limitations in its applications such as poor reproducibility, weak sensitivity and low automation (Sazuka et al., 2004). Isobaric tags for relative and absolute quantification (iTRAQ) is a robust mass spectrometry of protein quantitative technology, which can perform relative and absolute quantification in up to eight samples in parallel (Bindschedler and Cramer, 2011; Glibert et al., 2014). Recently, iTRAQ has been widely used for large-scale quantitative plant proteomic studies in exploration of various metabolic processes at the post-transcriptional level (Kambiranda et al., 2013; Martínez-Esteso et al., 2013) in response to various stresses (Yang et al., 2014; Li et al., 2015; Fu et al., 2016). Additionally, iTRAQ-based proteomics has been proven as a powerful method for unraveling the molecular regulatory networks involved in interactions between heavy metals and plant species, and a set of HM-responsive candidate proteins have been successfully identified. Alvarez et al. (2009) reported that exposure of *Brassica juncea* roots to cadmium (Cd) could activated several protein species involved in sulfur assimilation, redox homeostasis and xenobiotic detoxification, and depressed multiple proteins involved in protein synthesis and processing by two quantitative proteomics approaches including fluorescence two-dimensional differential gel electrophoresis (DIGE) and iTRAQ technology. More recently, the protein abundance changes in rice roots in response to Aluminum (Al) at an early phase were conducted with iTRAQ, and a total of 700 distinct protein species with >95% confidence were identified and 106 protein species were differentially accumulated upon Al toxicity in sensitive and tolerant cultivars (Wang et al., 2014). However, investigation of the dynamically protein abundance changes in response to Pb stress in radish has not been reported.

In this study, an iTRAQ-based quantitative proteomics approach was firstly employed to detect the effects of Pb responses at the protein abundance levels in radish roots. The differential abundance protein species (DAPS) involved in Pb-response of radish were quantified, and the enriched networks for regulating Pb stress at the protein level were acquired. Furthermore, to deeply reveal the integrative molecular network of radish plant response to Pb stress, the proteomic data were integrated with our previous transcriptomic, miRNA, degradome and metabolomic data, which provided a more global view of the molecular and cellular changes elicited by Pb stress in radish. This work would provide valuable information for further functional analyses of the critical Pb-responsive protein species in radish, which will be helpful for effectively facilitating the management of Pb and other HM contaminations in vegetable crops by genetic manipulation.

## Materials and methods

### Plant material

The variety of high-Pb-accumulation “NAU-RG” was selected for exploring the molecular regulation mechanisms in radish roots responding to the Pb stress. This genotype is an advanced inbred line with a medium size root in globular shape, white skin and flesh. According to the related evaluation methods and previous studies, different concentrations of Pb (NO_3_)_2_ (100, 200, 400, 500, 1000, and 1500 mg·L^−1^) were set to investigate the changes of visible physiological symptoms with different temporal durations in the preliminary experiment. Interestingly, no special obvious morphologic differences were found among individuals when exposed to low dose of heavy metal treatment, while the plants were seriously hampered and grew abnormally when exposed to 1500 mg·L^−1^ Pb (NO_3_)_2_. Therefore, the concentrations of Pb (NO_3_)_2_ at 500 mg·L^−1^ (Pb500) and 1000 mg·L^−1^ (Pb1000) were selected for the comparative proteomic analysis. Additionally, a control group was defined using non-treated seedlings (CK). The growth conditions of radish plants were conducted according to the reported descriptions (Wang et al., 2013). Plants were collected after 72 h with three different concentration treatments including an untreated control (CK) and two Pb-stressed conditions (Pb500 and Pb1000), based on the previous reported study (Wang et al., 2013). Each treatment consisted of three biological replicates. Equal amount of radish taproot samples (2.5 g) from three randomly selected individual plants of each replicate were pooled and cut into small pieces, which were rapidly frozen in liquid nitrogen and stored at −80°C for protein extraction.

### Protein extraction

Total protein was extracted from the radish taproots using a phenol (Phe) extraction procedure according to the reported methods with some modifications (Saravanan and Rose, 2004; Yang et al., 2013). In brief, about 2.5 g frozen taproots were finely powdered in liquid nitrogen adding 0.5 polyvinylpolypyrrolidone (PVPP) and then suspended in 20 mL of precooled extraction buffer containing 500 mM Tris-HCl pH 7.5, 50 mM ethylenediaminetetraacetic acid (EDTA), 100 mM KCl, 2 mM dithiothreitol (DTT), 2 mM phenylmethylsulfonyl (PMSF) and 30% sucrose (w/v). The mixture was extensively homogenized on ice for 2 min, and then an equal volume of Tris-HCl pH 7.5-saturated Phe was added. The mixture was thoroughly vortexed and proteins were collected by centrifuging at 12,000 g for 15 min at 4°C. The upper Phe phase was removed and re-extracted two or three times with extraction buffer. Proteins were precipitated from the final Phe phase with five volumes of saturated ammonium acetate in methanol at −20°C overnight. After centrifuging at 12,000 g for 15 min at 4°C, the pellets were washed twice with 20 mL of 0.1 mol·L^−1^ cooled acetone, and then dried by lyophilization and finally stored at −80°C until use. The protein was quantified by a Bradford protein assay kit (Sangon Biotech, China), and an equal amount of protein from three replicates of each treatment (CK, Pb500, and Pb1000) was respectively pooled for iTRAQ analysis.

### iTRAQ labeling and strong cation exchange (SCX) chromatography

The protein samples were dissolved in 100 mM triethylammonium bicarbonate (TEAB, pH 8.5) containing 1% SDS (w/v), reduced with 10 mM DTT at 56°C for 1 h, and followed by alkylation with 55 mM iodoacetamide (IAM) for 45 min at room temperature in the dark. Trypsin was then added to a final enzyme/substrate ratio of 1:20 (w/w) for protein digestion, which was incubated at 37°C for 12 h. The resulting tryptic peptides were vacuum-concentrated and then labeled with iTRAQ reagents (Applied Biosysterms, USA) according to manufacturer's instruction. The control sample (CK) was labeled with 118 iTRAQ reagent, and Pb-stressed samples treated with 500 and 1000 mg·L^−1^ Pb (NO_3_)_2_ were labeled with 119 and 114, respectively. The labeling reactions were incubated for 2 h at room temperature. Subsequently, all the peptides from three groups were combined and further fractionated using SCX chromatography on a Ultremex SCX column (250 × 4.6 mm, 5 μm particle size, 200 Å pore size, Phenomenex, USA) by high performance liquid chromatography (HPLC) system (Shimadzu LC-20AB, Japan). The HPLC gradient consisted of buffer A (25 mM NaH_2_PO4, 25% ACN, pH 2.7) for 10 min, 5−35% buffer B (25 mM NaH_2_PO_4_, 25% ACN, 1M KCL, pH 2.7) for 11 min and 35−80% buffer B for 1 min, which were eluted at a flow rate of 1 mL·min^−1^. The chromatograms were recorded at 214 nm and 20 constituents were obtained. The collected fractions were desalted with StrataX (Phenomenex, USA) and concentrated to dryness using a vacuum centrifuge.

### Liquid chromatography coupled with tandem mass spectrometry (LC-MS/MS)

The mass spectroscopy analysis was performed using an ABSCIE X TripleTOF 5600 mass spectrometer (AB SCIE X, Framingham, MA, USA), coupled with an online flow HPLC nanoAcquity system (Waters, USA), which comprised two parts of columns including Symmetry C18 column (5 μm particles, 180 um × 20 mm) and BEH130 C18 column specification (1.7 μm particles, 100 μm × 100 mm). Symmetry C18 column was used for adsorption and desalination of peptides, while BEH130 for the separation of peptides. The correction liquid (Thermo Fisher Scientific) was respectively added to liquid A (water:acetonitrile:formic acid = 98:2:0.1) and B (water:acetonitrile:formic acid = 2:98:0.1) with a certain proportion. A 2.25 μg aliquot of supernatant (9 μl) was transferred, and then the samples were eluted with liquid A at 2 μl·min^−1^ for 15 min for the purpose of adsorption and desalination of peptides. After that, the mobile phase which contain 5% liquid B was used to elute the supernatant (300 nl·min^−1^, 1 min). Next, the concentration of liquid B was raised from 5% to 35% in 40 min, from 35% to 80% in 5 min using linear gradients, and then the samples were eluted for 5 min with 80% liquid B. The separation was performed at a constant flow rate of 20 μl·min^−1^.

### Data analysis and protein identification

Raw data files were converted into MGF files using Proteome Discoverer 1.2. Proteins identification were performed by using Mascot search engine (Matrix Science, London, UK; version 2.3.02) against our sequenced radish root transcriptome database (SRX316199 and http://www.ncbi.nlm.nih.gov/sra/; Wang et al., 2013). The search parameters were set according to the published studies (Qiao et al., 2012; Yang et al., 2013; Wang et al., 2014). For protein identification, a mass tolerance of 0.05 Da was permitted for intact peptide masses and 0.1 Da for fragmented ions, with allowance for one missed cleavages in the trypsin digests. The methionine oxidation (M), protein N-terminal acetylation, deamidation (N, Q), and iTRAQ (Y) were selected as variable modifications, and carbamidomethyl modification of cysteines (C), iTRAQ (Nterminal), iTRAQ (K) were as fixed modifications. The search results were passed through additional filters before exporting the data. For protein identification, the filters were set as follows: significance threshold *P*, 0.05 (with 95% confidence) and ion score or expected cutoff <0.05 (with 95% confidence). For protein quantitation, it was required that a protein contains at least two unique spectra. The quantitative protein ratios were weighted and normalized by the median ratio in Mascot. The DAPS were defined: Showing a fold-change of greater than 1.2 or less than 0.83 using a greater statistically significant value *P* < 0.05.

### Functional analysis

The online software BLAST2GO (http://www.blast2go.com/b2ghome) was used to automatically assign protein description and obtain annotations from homologous sequences of public databases (Conesa et al., 2005; Conesa and Götz, 2008). A metabolic pathway analysis was undertaken based on the Kyoto Encyclopedia of Genes and Genomes (KEGG) pathway database. The Gene Ontology (GO) and metabolic pathway enrichment analysis of the DAPS were conducted through two publically available tools (webservers), namely, DAVID 6.7 and KOBAS 2.0. Furthermore, an integrated expression analysis of Pb-responsive miRNAs, mRNAs, metabolites and proteins were conducted based on our previous studies (Wang et al., 2013, 2015a,b). The same gene annotations were used to link expression levels across the four technologies.

## Results

### Protein identification and quantification of radish root in response to Pb stress

A total of 241,107 spectra were obtained from the iTRAQ LC−MS/MS proteomic analysis of these three group samples including an untreated control (CK), Pb500 and Pb1000. After data filtering to eliminate low-scoring spectra, a total of 17,579 unique spectra were matched to 10,896 peptides representing 9725 unique peptides. The criteria that the peptides with ≥ distinct unique peptide were used for protein identification, and a total of 3898 protein species were successfully detected at a 95% confidence limit. The relative molecular mass distribution of the identified protein species showed that the most abundant sequences ranged from 10 to 80 kDa.

**Figure 1:**
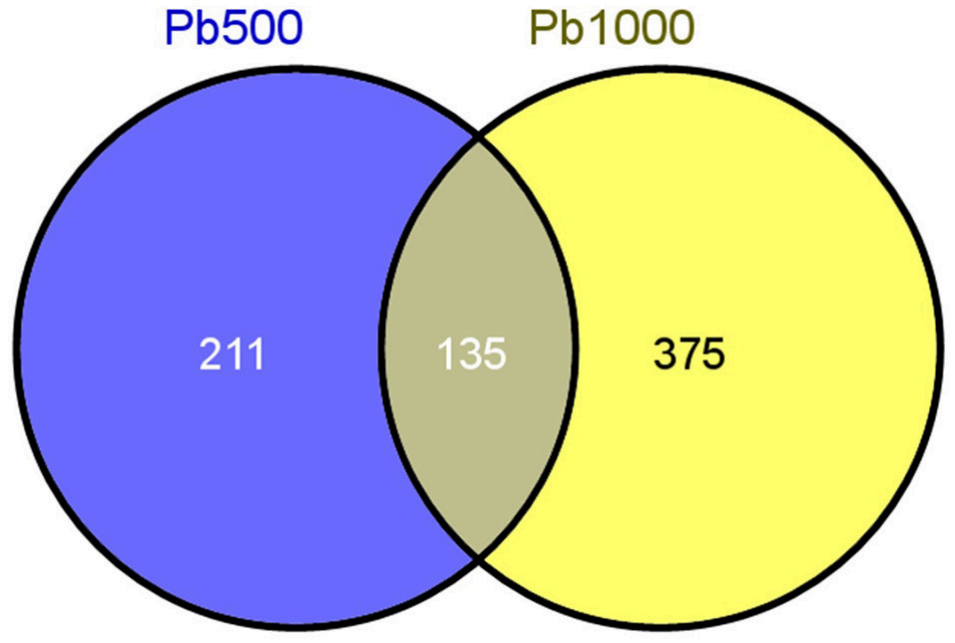
**The Venn diagram of differential abundance protein species (DAPS) involved in radish root under Pb500 and Pb1000 exposure**.

Only the proteins with at least two unique identified peptides were retained for further quantification analysis. In total, 2141 protein species were permitted the quantification of their abundance. Among them, 345 protein species were identified as differentially accumulated between Pb500 and CK groups including 177 (51%) up-accumulated, and 168 (49%) down-accumulated (Table S1). Among the 509 DAPS responding to the Pb1000 treatment, 48% (244) showed increased abundance, while 52% (265) showed decreased (Table S2). The proportion of common DAPS were calculated in response to the two Pb stress conditions. In total, 721 species were differentially accumulated under Pb treatments with 19% (135) common DAPS in both Pb treatments and 81% (586) distinct DAPS, among which 211 unique to Pb500-stress and 375 unique to Pb1000-stress (Figure 1).

### Functional classification and enrichment analysis of the DAPS

**Figure 2:**
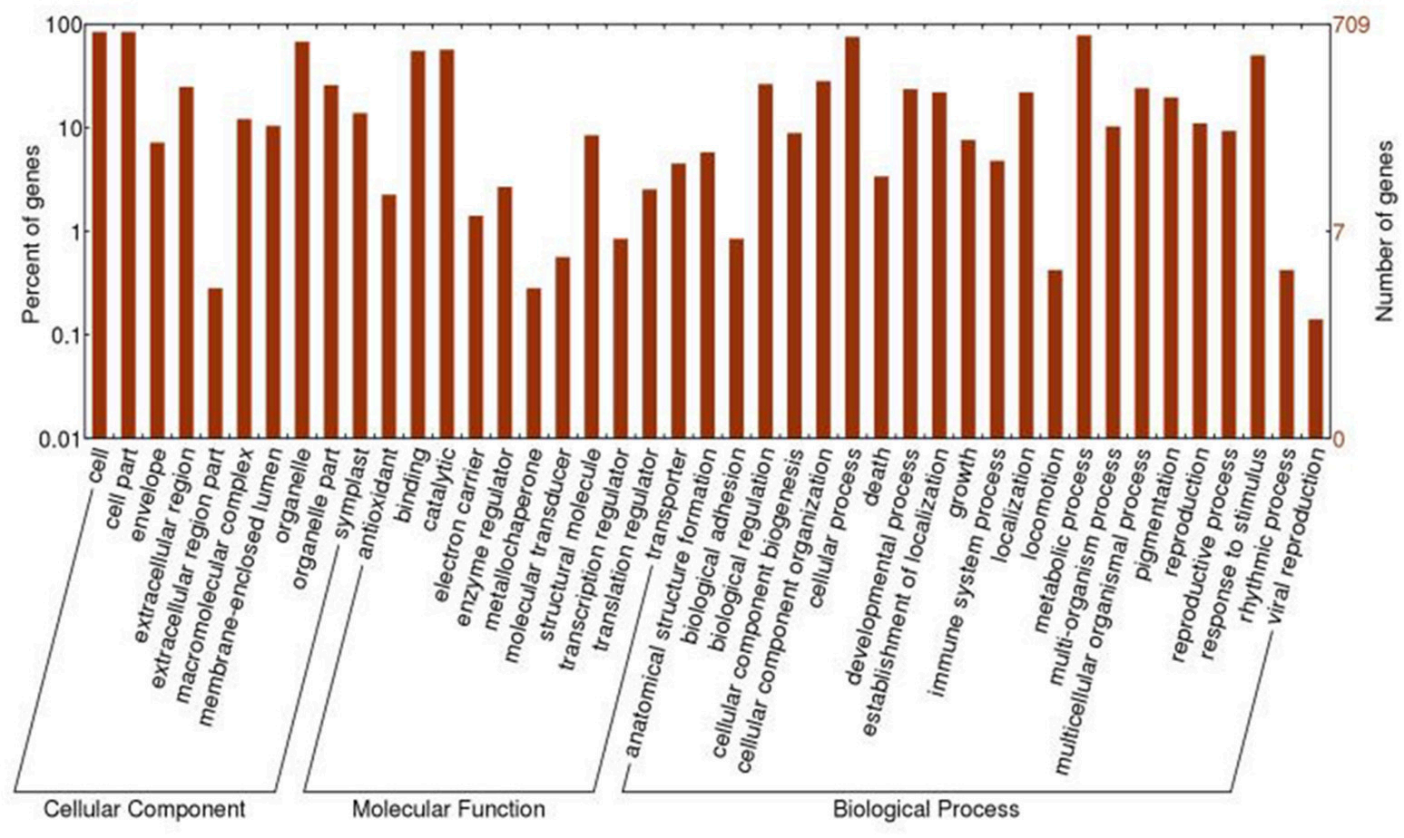
**GO classification of the DAPS of radish root in response to Pb stress**.

All of the 721 non-redundant DAPS sequences were functionally annotated using BLAST2GO (Conesa et al., 2005; Conesa and Götz, 2008). GO terms were assigned to query sequences and cataloged groups were produced basing on biological processes, molecular functions and cellular components. In total, 709 DAPS were assigned with 1687 GO terms and could be classified into 43 functional groups at the second level 2 (Figure 2). Among them, biological processes represented the largest category, containing 22 groups with metabolic (78.0%) and cellular process (75.3%) as the two most frequent terms. Within the molecular function category, the predominant groups were assigned to catalytic activity (56.7%) and binding (55.1%). For cellular components, all the DAPS were mostly located in cell (84.2%) and organelle (67.7%).

**Figure 3:**
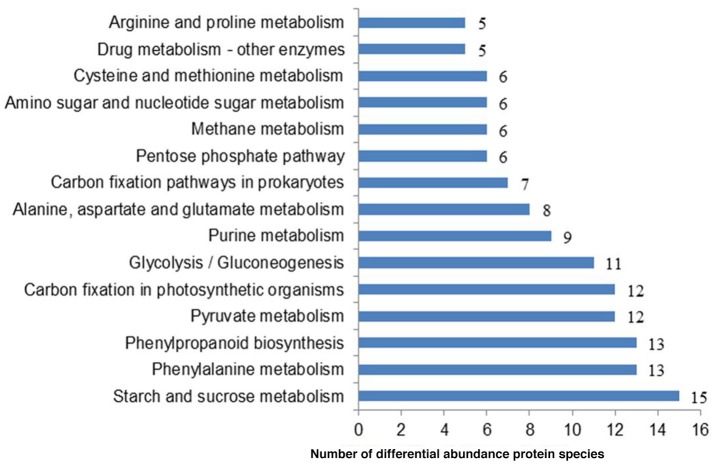
**The representative pathways of the DAPS involved in radish root response to Pb exposure**.

To systematically understand its biological functions in terms of networks, the 721 DAPS were mapped into the KEGG pathway database. A total of 75 pathways were assigned, which were largely involved in carbohydrate metabolism (such as starch and sucrose metabolism, pyruvate metabolism, glycolysis/gluconeogenesis, pentose phosphate pathway, and amino sugar and nucleotide sugar metabolism), amino acid metabolism (phenylalanine metabolism, alanine, aspartate, and glutamate metabolism, cysteine and methionine metabolism and arginine and proline metabolism) and energy metabolism (carbon fixation in photosynthetic organisms, carbon fixation pathways in prokaryotes, methane metabolism, oxidative phosphorylation and sulfur metabolism) (Table S3). The pathways with the DAPS number larger than five were shown in Figure 3.

Furthermore, through functional enrichment analysis of the 135 common DAPS (Table 1), it was shown that “cell wall” (GO: 0005618), “apoplast” (GO: 0048046), “response to metal ion” (GO: 0010038), “vacuole” (GO: 0005773), and “peroxidase activity” (GO: 0004601) were the most overrepresented GO terms (Table 2). KOBAS 2.0 webserver was employed to identify statistically enriched pathways from pathway databases involved in KEGG, BioCyc, Reactome, Pathway Interaction and Panther. The critical enriched pathways included “citric acid (TCA) cycle and respiratory electron transport,” “pyruvate metabolism,” “phenylalanine metabolism,” “phenylpropanoid biosynthesis,” and “carbon metabolism” (Table 3).

**Table 1:** **The common DAPS involved in radish root under Pb500 and Pb1000 exposure**.

**Accession**	**Protein ID**	**Description**	**Pb500/CK**	**Pb1000/CK**
**PROTEIN METABOLISM**				
1	CL10682.Contig1	20s proteasome subunit	1.508	0.729
2	CL6704.Contig2	40s ribosomal protein	0.758	0.781
3	CL8114.Contig2	60s acidic ribosomal protein	1.452	1.850
4	Unigene19731	60s acidic ribosomal protein	1.599	1.448
5	CL2397.Contig1	60s ribosomal	1.267	1.708
6	Unigene1307	60s ribosomal protein	0.749	0.616
7	CL9354.Contig1	60s ribosomal protein	0.625	1.255
8	CL10582.Contig2	60s ribosomal protein	2.084	3.621
9	CL2279.Contig3	60s ribosomal protein	1.404	3.372
10	CL5126.Contig2	60s ribosomal protein	1.377	2.232
11	Unigene2385	Cyclase family protein	0.675	0.555
12	Unigene21215	Cyclophilin 1	1.430	0.413
13	CL714.Contig1	d-3-phosphoglycerate chloroplastic-like	0.654	1.524
14	CL89.Contig1	Eukaryotic translation initiation factor	1.214	1.258
15	Unigene29764	RNA binding protein	1.298	1.715
16	CL1044.Contig6	RNA helicase drh1	0.735	0.632
17	CL7993.Contig7	RNA-binding protein	1.568	2.112
18	CL3437.Contig4	Root hair defective 3	0.820	1.512
19	Unigene19929	Rotamase cyclophilin 2	1.203	0.485
20	CL14184.Contig1	Sar DNA-binding protein	1.555	3.115
21	CL9725.Contig1	Small nuclear ribonucleoprotein g	0.789	0.805
22	Unigene926	Translation initiation factor	1.842	1.386
23	Unigene26860	Translation initiation family protein	1.496	1.660
24	CL4590.Contig3	Ubiquitin-conjugating enzyme e2 5	1.502	0.606
25	CL83.Contig2	Ubiquitin-conjugating enzyme e2 variant 1d	1.204	0.772
26	Unigene282	Ubiquitin-conjugating enzyme e2-17 kda	1.292	0.717
27	CL1707.Contig1	protein Disulfide isomerase	1.254	1.339
**STRESS AND DEFENSE**				
28	CL14023.Contig1	Profilin 1	1.487	0.731
29	Unigene29769	Leucine-rich repeat family protein	0.509	0.539
30	CL9570.Contig1	Leucine-rich repeat receptor-like protein	0.759	0.423
31	CL7251.Contig1	Temperature-induced lipocalin	0.477	0.710
32	Unigene29834	Thaumatin-like protein	0.112	0.782
33	Unigene8320	Disease resistance response	0.652	0.410
34	Unigene29734	Early nodulin-93-like	0.535	0.548
35	CL6279.Contig2	Early nodulin-like protein 13	2.053	1.928
36	CL752.Contig1	Blue copper-binding 15k	1.315	0.446
37	CL77.Contig1	Chitinase family protein	0.762	0.561
38	Unigene16718	Lectin-like protein	0.606	0.562
39	CL7174.Contig1	Peroxidase	0.743	0.438
40	CL1027.Contig4	Peroxidase	0.567	0.542
41	Unigene8343	Peroxidase	1.318	0.602
42	Unigene21004	Peroxidase 12	0.520	0.548
43	Unigene21694	Peroxidase atp3a homolog	1.228	0.331
44	CL3864.Contig1	Peroxidase atp8a	0.791	0.679
45	Unigene27310	atpca atprx33 prx33	0.374	0.377
46	CL959.Contig2	Bacterial-induced peroxidase	0.831	0.656
47	CL13300.Contig1	Thioredoxin 1	0.714	0.764
48	CL2689.Contig1	12-oxophytodienoate reductase 1	1.545	1.274
49	CL5307.Contig5	12-oxophytodienoate reductase 3	0.765	0.566
50	CL10604.Contig2	Glutaredoxin-like protein	1.547	1.752
51	CL4589.Contig1	Anamorsin homolog	0.788	0.416
52	CL11999.Contig2	Monodehydroascorbate reductase	0.823	1.456
53	CL2553.Contig1	Adenine nucleotide alpha hydrolases-like superfamily protein	0.682	1.376
54	CL11402.Contig1	Reversibly glycosylated polypeptide-3	1.417	1.784
55	CL2995.Contig1	Selenium-binding protein	0.663	0.661
56	CL8292.Contig1	Serpin family protein	0.716	0.625
57	CL214.Contig1	Strictosidine synthase	0.668	0.658
58	CL2053.Contig1	Stromal cell-derived factor 2-like protein	1.383	1.618
59	Unigene15048	Subtilase family protein	0.653	0.568
60	Unigene6675	Subtilisin-like protease-like	0.662	0.445
61	CL7173.Contig1	Thiol protease aleurain	1.940	1.247
62	CL603.Contig2	tpx2 (targeting protein for xklp2)-like protein	2.121	1.589
**SIGNAL TRANSDUCTION AND TRANSPORT**				
63	CL14365.Contig1	Elongation factor 1-alpha	1.519	2.972
64	CL11865.Contig1	Receptor kinase	0.664	0.650
65	CL292.Contig1	Signal recognition particle 72 kda	0.677	1.252
66	Unigene8449	cdp-diacylglycerol–glycerol-3-phosphate 3- partial	0.792	0.584
67	CL2540.Contig2	rae1-like protein at1g80670-like	1.566	0.644
68	CL1217.Contig1	Clathrin light chain protein	1.860	1.231
69	CL10110.Contig2	Calreticulin-3-like isoform x1	0.648	2.147
**CARBOHYDRATE AND ENERGY METABOLISM**				
70	CL3177.Contig3	Aldose 1-epimerase family protein	0.650	0.485
71	CL12533.Contig1	ATP citrate lyase	1.214	1.424
72	Unigene22436	Beta-fructofuranosidase 5	1.625	0.726
73	Unigene24884	Beta-glucosidase 21	1.993	2.132
74	CL13638.Contig1	Electron transfer flavoprotein subunit mitochondrial-like	1.278	0.615
75	Unigene27210	Fumarate hydratase 1	0.771	0.679
76	Unigene20278	Fumarate hydratase mitochondrial-like	0.671	0.569
77	CL10499.Contig1	Glucan endo- -beta-glucosidase 4	1.252	0.441
78	Unigene25466	Glucan endo- -beta-glucosidase 6-like	1.268	0.659
79	CL4038.Contig1	Isocitrate dehydrogenase	0.803	0.743
80	Unigene23018	Malate dehydrogenase	0.778	0.735
81	CL8917.Contig1	Myosin heavy chain kinase	1.319	1.551
82	Unigene28617	NADH-cytochrome b5 reductase-like	1.244	0.666
83	CL6783.Contig1	Pyruvate dehydrogenase family protein	0.724	0.819
84	CL6151.Contig2	Protein weak chloroplast movement under blue light 1-like	1.657	2.037
85	CL5706.Contig6	Transketolase	1.426	5.285
86	CL859.Contig3	Transaldolase-like protein	0.808	1.288
87	CL5075.Contig1	Succinate-semialdehyde mitochondrial-like isoform x1	0.790	0.811
88	CL3677.Contig1	Aconitase c-terminal domain-containing protein	1.266	0.805
89	CL452.Contig1	Beta-xylosidase alpha-l-arabinofuranosidase 2-like	0.524	0.526
90	CL452.Contig4	Beta-xylosidase alpha-l-arabinofuranosidase 2-like	0.754	0.451
91	CL7567.Contig2	Dihydrolipoyl dehydrogenase 2	0.705	1.526
**AMINO ACID AND LIPIDS METABOLISM**				
92	Unigene10784	Macrophage migration inhibitory factor family protein	1.567	0.560
93	Unigene24925	gdsl esterase lipase at1g54790-like	2.379	0.683
94	Unigene8067	gdsl esterase lipase at3g26430-like	0.540	0.450
95	Unigene22679	Glycine dehydrogenase	0.787	0.768
96	Unigene25700	Glycosyl hydrolase family 38 protein	0.738	0.572
97	Unigene24148	Xylem cysteine proteinase 1-like	0.811	0.566
98	CL9552.Contig2	Methyltransferase pmt24	1.336	1.571
99	Unigene2767	Myosin-related family protein	1.327	1.358
100	CL12773.Contig2	Nascent polypeptide-associated complex subunit alpha-like	1.602	0.562
101	CL2541.Contig2	Serine carboxypeptidase-like 29	3.161	1.950
102	Unigene2790	Aminomethyltransferase	0.601	0.585
103	CL783.Contig2	Peptidyl-prolyl cis-trans isomerase cyp20-3	0.566	0.629
104	CL2368.Contig1	Proteasome subunit alpha type-2-b	1.292	0.574
105	Unigene289	s9 Tyrosyl aminopeptidase	1.606	2.699
106	CL6065.Contig3	Aspartic proteinase	0.511	0.612
107	CL9279.Contig1	Aspartyl protease family protein	0.503	0.179
108	CL5981.Contig1	Dimethylmenaquinone methyltransferase family protein	1.301	0.408
109	CL330.Contig1	Cupin family protein	0.714	0.522
110	CL2774.Contig2	Dehydrin erd14	1.678	1.539
**CELL WALL AND CYTOSKELETON**				
111	CL2803.Contig4	Polygalacturonase inhibiting protein	0.383	0.491
112	CL12269.Contig1	Polygalacturonase-like protein	0.703	0.502
113	CL1120.Contig1	GTP-binding protein sar1a-like	1.252	1.646
114	CL741.Contig1	Nuclear RNA binding	1.847	1.539
115	CL14456.Contig1	Neurofilament protein	3.295	1.200
116	CL5177.Contig3	Nuclear protein	1.503	2.413
117	CL319.Contig1	Nucleic acid binding isoform partial	1.319	1.582
118	CL12221.Contig1	Nucleolin family protein	1.464	1.674
119	CL1396.Contig2	Nucleolin like 1	1.550	1.814
120	CL4932.Contig1	Pectinacetylesterase family protein	1.306	0.638
121	Unigene6860	Pectinesterase inhibitor	0.630	0.563
122	CL2232.Contig2	Probable pectinesterase pectinesterase inhibitor 51-like	0.823	0.627
123	CL1396.Contig2	Nucleolin like 1	1.550	1.814
124	Unigene21122	GPI-anchored protein	0.695	0.420
125	CL2657.Contig1	Lipid-associated family protein	1.459	0.475
126	Unigene25152	Vesicle associated protein	1.618	1.678
127	Unigene22796	Organellar DNA-binding protein 1	0.756	0.544
128	CL8050.Contig1	Partial	1.295	0.681
129	Unigene2988	O-glycosyl hydrolases family 17 protein	1.529	0.726
130	Unigene29720	Endochitinase isolog	0.619	0.651
131	Unigene29711	Adenine nucleotide alpha hydrolases-like protein	0.698	0.472
132	CL3373.Contig1	Luminal binding protein	1.210	1.573
**UNCHARACTERIZED PROTEIN**				
133	CL12123.Contig1	Unknown	3.464	1.979
134	CL8554.Contig2	Uncharacterized protein	1.310	0.677
135	CL14500.Contig1	Uncharacterized protein	1.643	0.763

**Table 2:** **The dominant enriched GO terms for the 135 common DAPS under Pb500 and Pb1000 exposures**.

**Go term**	**ID**	**Input number**	**Background number**	***P*-Value**	**Corrected *P*-Value**
Cell wall	GO:0005618	24	480	4.69E-12	2.89E-09
Apoplast	GO:0048046	18	267	2.21E-11	9.07E-09
Response to metal ion	GO:0010038	17	425	1.25E-07	2.77E-05
Vacuole	GO:0005773	21	650	1.42E-07	2.77E-05
Peroxidase activity	GO:0004601	8	68	1.62E-07	2.77E-05
Plasmodesma	GO:0009506	20	645	5.16E-07	5.43E-05
Organelle lumen	GO:0043233	15	373	6.29E-07	5.54E-05
Membrane-enclosed lumen	GO:0031974	15	376	6.93E-07	5.69E-05
Antioxidant activity	GO:0016209	8	86	8.46E-07	6.51E-05
Response to inorganic substance	GO:0010035	25	1085	4.04E-06	0.000293
Cytosol	GO:0005829	27	1270	7.07E-06	0.000484
Nucleolus	GO:0005730	10	208	1.04E-05	0.000677
Extracellular region	GO:0005576	40	2386	1.48E-05	0.00091
Plant-type cell wall	GO:0009505	10	240	3.37E-05	0.001442
Positive regulation by symbiont of host innate immune response	GO:0052166	3	6	3.86E-05	0.001442

**Table 3:** **The dominant pathways for the 135 common DAPS under Pb500 and Pb1000 exposure**.

**Term**	**Database**	**Input number**	**Background number**	***P*-Value**	**Corrected *P*-Value**
The citric acid (TCA) cycle and respiratory electron transport	Reactome	5	26	2.30E-05	0.001287
Citrate cycle (TCA cycle)	KEGG PATHWAY	7	60	0.000112	0.002884
Phenylalanine metabolism	KEGG PATHWAY	9	106	0.000117	0.002933
Pyruvate metabolism and Citric Acid (TCA) cycle	Reactome	4	22	0.000192	0.004371
Phenylpropanoid biosynthesis	KEGG PATHWAY	10	145	0.000243	0.005078
Citric acid cycle (TCA cycle)	Reactome	3	10	0.000369	0.007341
Carbon metabolism	KEGG PATHWAY	11	217	0.001399	0.022388

### Characterization of the critical Pb stress-responsive proteins in radish

Protein modification, the balance between synthesis and degradation, is a critical form of regulation that is coordinated to achieve a unified cellular under the stress of environmental stimuli (Hinkson and Elias, 2011). In this study, 27 protein species implicated in protein translation, processing and degradation were identified to be co-differentially accumulated during Pb500 and Pb1000 exposures. Among them, nine ribosomal protein species were up-accumulated under either one or both Pb treatments, while only one ribosomal component was both down-accumulated during the two Pb-stressed conditions. The abundance levels for three translation initiation family proteins (CL89.Contig1, Unigene926 and Unigene26860), two RNA binding proteins (Unigene29764 and CL7993.Contig7) and one protein disulfide isomerase (CL1707.Contig1) were observed to be increased under these two Pb exposures. However, one RNA helicase (CL1044.Contig6) was identified to be decreased responding to these two Pb treatments. All three identified ubiquitin-conjugating enzymes (CL4590.Contig3, CL83.Contig2, and Unigene282) and one cyclase family protein (Unigene2385) were coordinately increased at the Pb500 exposure while decreased at the Pb1000 (Table 1).

It is known that the antioxidant enzymes could ensure cellular protection from the reactive oxygen species (ROS) mediated damage in plant responding to various environmental stresses. In the present study, the abundance changes for many antioxidant enzymes were detected including two 12-oxophytodienoate reductases (CL2689.Contig1 and CL5307.Contig5), one glutaredoxin-like protein (CL10604.Contig2), one thioredoxin (CL13300.Contig1) and eight peroxidases (CL7174.Contig1, CL1027.Contig4, Unigene8343, Unigene21004, Unigene21694, CL3864.Contig1, Unigene27310, and CL959.Contig2). Among these identified DAPS for antioxidant enzymes, most of them were decreased in radish under the exposure of Pb except one OPR (CL2689.Contig1) and one GR (CL10604.Contig2) up-accumulated at both Pb treatment, and two peroxidases (Unigene 8343 and Unigene 21694) up-accumulated under the Pb500 exposure (Table 1).

Additionally, a lot of protein species involved in carbohydrate and energy metabolism-related pathways (i.e., “TCA cycle and respiratory electron transport,” “pyruvate metabolism,” and “carbon metabolism”) were shown to exhibit abundance changes under Pb exposure. The activities of most metabolic enzymes involved in these pathways were both repressed in the two concentrations of Pb treatments, including malate dehydrogenase (Unigene23018), endochitinase (Unigene29720), lectin-like protein (Unigene16718), polygalacturonase-like protein (CL12269.Contig1), aldose 1-epimerase family protein (CL3177.Contig3), beta-xylosidase alpha-l-arabinofuranosidase 2-like (CL452.Contig1), chitinase family protein (CL77.Contig1), and glycosyl hydrolase family 38 protein (Unigene25700). However, other important enzymes showed differentially accumulated during the two Pb stresses. For example, the expression levels of the acid invertase beta-fructofuranosidase 5 (Unigene22436), a beta-glucosidase glucan endo-beta-glucosidase 4 (CL10499.Contig1), and a hydrolases o-glycosyl hydrolases family 17 protein (Unigene2988) were increased in Pb500 exposure, but decreased in Pb1000 stress (Table 1).

### Associated analysis of Pb-responsive genes through proteomic in combination with other–omic techniques of radish in response to Pb stress

In order to investigate whether the protein level is correlated with the corresponding mRNA level alterations, the proteomic data was compared with our previous transcriptome data between CK vs. Pb1000 (Wang et al., 2013). Proteins were considered to be correlated when quantified proteins have expression information at the transcriptome level, and a total of 1667 protein species (77.9% of all the quantified proteins) were detected from the transcriptome data (Table S4). Out of the 510 protein species found to be significantly accumulated under Pb1000 stress of radish, there were only 57 DAPS could be matched with their cognate differentially expressed genes (DEGs) including 25 regulated in the same trends and 32 found in the opposite direction (Table S5). In addition, there were 343 DAPS exhibiting no change in mRNA expression and 144 DEGs without altered expression at protein level (Table S5). These results indicated that the accumulation of transcripts and proteins occur independently. A generally low congruency of proteomic and transcriptional profiles has been reported in other previous studies (Lan et al., 2011; Zhuang et al., 2013).

Increasing evidences have revealed that miRNA-mediated gene regulation play significant roles in plant response to HM stress, and 19 Pb-responsive miRNAs and their corresponding target mRNAs were identified in radish by siRNA sequencing and degradome analysis (Wang et al., 2015a). By exploring an integrated expression analysis of miRNA, mRNAs and proteins, a total of seven miRNA-mRNA pairs and matching proteins were identified in radish response to Pb stress, which mainly included two miRNA families, miR156 and miR396. As shown in Table 4, almost all Pb-responsive miRNAs and their corresponding mRNA targets had an anti-relationship at protein level. For example, two members of miR156 family (miR156b and miR156c) were found to be up-regulated in response to Pb exposure, while the expressions of their targets (glutaredoxin, aldose 1-epimerase and malate dehydrogenase) were all repressed at protein levels. Two corresponding protein species for the down-regulated miR396b were identified during Pb stress of radish, one (Translation initiation factor eIF-3) was up-accumulated, while the other one (thioredoxin-like 1-2, chloroplastic) was shown to be down-accumulated.

**Table 4:** **Association analysis of miRNAs, genes, and proteins responsive to Pb stress in radish root**.

**miRNA**	**LogFC^a^**	**Target mRNA annotation**	**Protein ID**	**LogFC^b^**	**LogFC^c^**
miR156b	1.1813	Glutaredoxin	CL10604.Contig2	−0.12	−0.247
miR156b	1.1813	Aldose 1-epimerase	CL3177.Contig3	−0.19	−0.314
miR156c	1.0788	Malate dehydrogenase	Unigene23018	−0.11	−0.134
miR396b	−0.5131	Thioredoxin	CL13300.Contig1	−0.15	−0.117
miR396b	−0.5131	Translation initiation factor	CL89.Contig1	0.084	0.0997
miR396b	−0.5131	Translation initiation factor	Unigene926	0.265	0.1418
miR396b	−0.5131	Translation initiation factor	Unigene26860	0.175	0.2201

a *miRNA fold change under Pb500*.

b *Protein fold change under Pb500*.

c *Protein fold change under Pb1000*.

For deeply dissecting the molecular mechanism underlying Pb tolerance and homeostasis in radish, we further performed an association analysis of the seven Pb-responsive miRNA-mRNA-proteins pairs with the differentially regulated metabolites identified in radish roots during Pb stress (Wang et al., 2015b). Based on the intersected pathway analysis, three Pb-responsive metabolites including glucose, citrate and malate were found to be linked with the miRNA-mRNA-proteins, which may play a significant role in radish response to Pb stress (Table S6).

## Discussion

Proteomic technique provides a powerful tool for the analysis of molecular mechanism of plant response against stresses, and a path for linking gene expression to cell metabolism in the rapidly processing post-genome era (Ahsan et al., 2009; Liang et al., 2013). Increasing evidences have revealed that proteomic studies are playing important roles in the post-genomic era for characterizing the molecular mechanisms underlying plant responses to HM stresses (Fukao et al., 2011; Wang et al., 2013, 2014; Sebastiani et al., 2014). Radish is an important root vegetables worldwide, which has been considered as one of the most significant root species for dissecting the molecular regulatory network of HM stress in Brassicaceae crops (Xu et al., 2012; Wang et al., 2014, 2015a; Liu et al., 2015; Xie et al., 2015). In the current study, comparative proteomics analysis by iTRAQ together with other-omics techniques reveals complex regulatory network and provides insights into the response of radish root under Pb exposure. To the best of our knowledge, this is the first study to systematically investigate and characterize the protein abundance changes under Pb stress exposure in radish root with iTRAQ-based proteomics analysis.

### Proteins of signal sensing mechanisms involved in radish root response to Pb stress

It was known that the cell wall could activate a variety of specific stress-responsive signaling proteins when perceive outer stress conditions (Jamet et al., 2006; Day et al., 2013). In previous studies, a set of cell wall-related protein species exhibited dynamic changes in response to HM stress, such as Al exposure in rice and cadmium (Cd) exposure in flax (Hradilová et al., 2010; Wang et al., 2013, 2014). Currently, “cell wall” (GO: 0005618) was identified as the most enriched GO term for all the 135 common DAPS involved in the Pb500 and Pb1000 stress, which indicated that cell wall may play a vital role in response to Pb stress of radish. Two critical inhibiting protein species including a pectinesterase inhibitor (CL2232.Contig2) and a polygalacturonase inhibitor (CL2803.Contig4) were both down-accumulated during the stresses of Pb500 and Pb1000 in radish. The two protein species were verified to modulate the activities of pectinesterases (PE) and polygalacturonases (PG), which could help the plant to fine-tune cell wall remodeling processes when exposure stress conditions (Pelloux et al., 2007; Ferrari et al., 2012). The down-accumulation of the inhibiting proteins maybe result in up- accumulation of PE and PG, indicating their positive role to the cell wall remodeling when encounter the Pb stress of radish.

Calreticulin (CRT) is an abundant endo reticulum Ca^2+^ binding protein, and plays a critical role in Ca^2+^ homeostasis and signaling sensing network (Corbett and Michalak, 2000; Jia et al., 2009). In this study, one CRT (calreticulin-3-like isoform x1, CL10110.Contig2) was found to be induced by the Pb1000, indicating that the altered expression of CRT maybe function as signaling molecular in modulating the radish plant to adapt to the Pb-stressed environments. Additionally, jasmonate (JA) is known to be a vital signaling molecule which can be activated in response to a wide range of environmental cues including heavy metals (Maksymiec et al., 2005; Schommer et al., 2008; Liu et al., 2009). In this study, the expression level of 12-oxo-phytodienoic acid reductase (OPR), a key enzyme involved in the JA biosynthesis pathway, showed differential abundance levels during the Pb stress. The OPR-1 (CL2689.Contig1) was up-accumulated in radish roots when subjected to the either Pb500 or Pb1000 stress, and a similar result was found at the proteome level in rice root exposure to As stress (Ahsan et al., 2008; Srivastava et al., 2009). However, the expression level of OPR-3 (CL5307.Contig5) was decreased in radish roots under the conditions of Pb500 and Pb1000 exposure, indicating that different members of the OPR were differentially accumulated under the Pb exposure in plant.

### Proteins of carbohydrate and energy metabolism involved in radish root response to Pb stress

The accumulation of HM in plants can severely affect the photosynthetic pathway and thus lead to symptoms of toxicity, such as chlorosis and growth reduction (Pourrut et al., 2013). To maintain the normal growth and development, or at least to protect the cells against excess damages, plants need to activate the carbohydrate and energy metabolism related metabolic pathways (Sarry et al., 2006; Thapa et al., 2012). In this study, “citric acid (TCA) cycle and respiratory electron transport” was identified as the most enriched pathway, and a lot of critical metabolic enzymes were shown to exhibit abundance changes under Pb exposure. Beta-glucosidase catalyzes the hydrolysis of the glycosidic and release of glucose, which then entry into the glycolysis process. ATP-citrate lyase (ACL, EC4.1.3.8) is a key enzyme involved in TCA cycle, which catalyzes the cleavage of citrate to yield acetyl CoA, oxaloacetate, ADP, and orthophosphate, and the ACL gene was found to be induced under various stresses including low temperature and drought stimulus (Hu et al., 2015). Pyruvate dehydrogenase could exert a key role in linking the glycolysis to the TCA cycle by catalyzing the formation of an acetyl-CoA from pyruvate (Vuoristo et al., 2015), which is a vital rate-limiting step reaction that determines the rate and efficiency of TCA cycle. In this study, a beta-glucosidase 21 (Unigene24884), ATP citrate lyase (ACL12533.Contig1), and a pyruvate dehydrogenase (CL6783.Contig1) were observed to be both up-accumulated during the Pb500 and Pb1000 stress of radish root, which revealed their positive potential role in producing more reducing power to compensate high-energy demand response to Pb stress. However, the abundances of the some other metabolic enzymes including Isocitrate dehydrogenase (CL4038.Contig1), fumarate hydratase (Unigene27210, Unigene20278), and malate dehydrogenase (Unigene23018) were decreased under the Pb-stresses, indicating their activity suffered repression. These decreased proteins may influence the biosynthesis and accumulation of organic acids such as citrate and malate, which play critical roles in HM tolerance (Ma et al., 2001; Wang et al., 2015b).

### Proteins of antioxidative defense and detoxification involved in radish root response to Pb stress

The presence of excess metal ions causes ROS in plants, which can irreversibly damage the cells and attack macromolecules (Ahsan et al., 2009). However, the ROS can be scavenged by plant antioxidant defense system consisting of antioxidant compounds and enzymes (Apel and Hirt, 2004; Suzuki et al., 2012). The ascorbate (ASA)–glutathione (GSH) cycle is one of the main antioxidant systems in plants to keep ROS under control being, which involves a lot of critical antioxidant enzymes in a series of cyclic reactions to detoxify H_2_O_2_ (de Sousa et al., 2016; Noshi et al., 2016). One of the antioxidant enzymes is peroxidase (POD), which can detoxify H_2_O_2_ by oxidizing ascorbate. It was reported that different members of the peroxidase gene family were differentially accumulated under various HM exposures in Arabidopsis (Kumari et al., 2008), wheat (Houde and Diallo, 2008) and aspen (Grisel et al., 2010). In this study, the term of “peroxidase activity” (GO: 0004601) was identified as one of the most over-represented GO terms and six POD protein species were identified as DAPS, indicating the POD may be the critical ROS-scavenging protein in radish root response to Pb stress. The abundance of another antioxidant enzyme involved in ASA–GSH cycle, monodehydroascorbate reductase (MDHAR, CL11999.Contig2) was also found to be altered in response to Pb stress. The main function of MDHAR is responsible for ascorbate regeneration in plant tissues, and the expression level changed during the HM stress in *B*. *juncea* root (Alvarez et al., 2009) and *A*. *Halleri* shoot (Farinati et al., 2009). Additionally, many thiol-containing antioxidants, peroxiredoxin (PRX33, unigene27310), glutaredoxin-like protein (GRX, CL10604.Contig2) and thioredoxin (TRX, CL13300.Contig1), were also found to be altered during the Pb-stressed condition of radish root. The Prx is a thiol peroxidase with multiple functions, which was found to be induced under various HM stresses such as Cd (Hossain et al., 2013) and As (Pandey et al., 2012). Glutaredoxin (GRX) and thioredoxin (TRX) could be oxidized by peroxides and regenerate peroxiredoxins, which were verified to play direct roles in the antioxidative system by regenerating peroxiredoxins oxidized by peroxides (Hossain and Komatsu, 2013).

### The genetic regulatory network of radish root response to Pb stress

**Figure 4:**
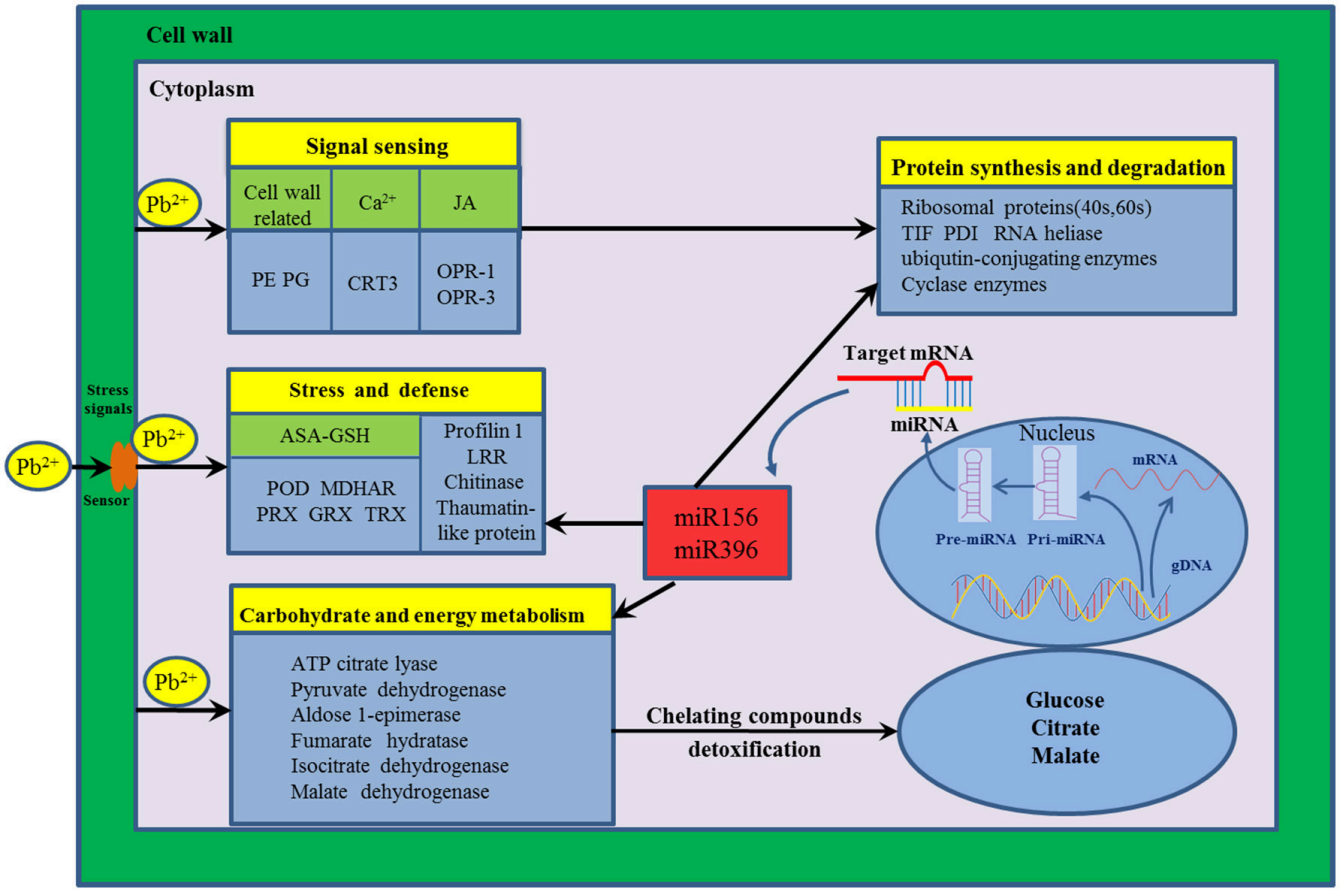
**The putative genetic regulatory network of Pb stress response in radish root**. 12-oxo-phytodienoic acid reductase-1 (OPR-1), 12-oxo-phytodienoic acid reductase-3 (OPR-3), Ascorbate (ASA)–Glutathione (GSH), Calreticulin-3-like isoform x1 (CRT3), Glutaredoxin (GRX), thioredoxin (TRX), Leucine-rich repeat family protein (LRR), Monodehydroascorbate reductase (MDHAR), Pectinesterases (PE), Peroxidase (POD), Peroxiredoxin (PRX), Polygalacturonases (PG), Protein disulfide isomerase (PDI), Translation initiation factor (TIF).

The mechanism of HM response is a complex process that a variety of genes and response components involved in plants (Fukao et al., 2011; Sebastiani et al., 2014). In the present study, to reveal the molecular mechanism underlying Pb stress response in radish root, a putative schematic network was put forward based on proteomic information of this study in conjunction with integrated analysis of miRNA, transcriptomic and metabolomic data (Figure 4). Once Pb enters the cell of radish plants, the cell wall firstly perceive stress signals and then activate specific stress-responsive signaling molecules including fine-tuning cell wall remodeling processes through special proteins like PE and PG, triggering the HM stress responsive hormones levels such as JA as well as modulating calcium-signaling molecules. With the aid of signaling molecules, the stress signals were transmitted and ultimately give rise to the alterations in gene expression and protein levels. A direct consequence of HM stress was the disturbance in the balance of protein synthesis and degradation, which is essential to both cellular homeostasis and dynamics because almost all biological processes need the involvement of enzymes. Under Pb stress, many key enzymes (i.e., ATP citrate lyase, Isocitrate dehydrogenase, fumarate hydratase and malate dehydrogenase) involved in the glycolysis and TCA cycle were severely affected, which ultimately cause alteration of some metabolites including glucose, citrate and malate. The glucose could act as the osmoprotectants protecting the cell constituents, and the organic acids (such as citrate and malate) played critical roles in chelating toxic HM ions. Meanwhile, a series of other defense responses were triggered to cope with Pb-induced injuries. For example, the ASA–GSH cycle was the main antioxidant systems for scavenging the accumulated ROS to alleviate oxidative damages. Moreover, several Pb-defense protein species (glutaredoxin, aldose 1-epimerase malate dehydrogenase and thioredoxin) encoding genes targeted by miRNA156 and miR396, which were found to play critical roles in defense system when radish root responds to Pb stress.

In summary, this is the first report on systematically investigating and characterizing the protein abundance changes under Pb stress in radish root with the iTRAQ technique. A total of 3898 protein species were successfully detected and 2141 were quantified. A subset of 721 protein species were differentially accumulated upon at least one Pb exposure, and 135 ones showed common abundance alterations under two Pb-stressed conditions. GO and pathway enrichment analysis revealed that these 135 common DAPS were strongly enriched in the categories of cell structure, carbohydrate and energy metabolism-related pathways and antioxidative defense. Furthermore, the integrative analysis of transcriptomic, miRNA, degradome and metabolomic with proteomic data provided a strengthened understanding of radish root response to Pb stress, and a schematic genetic regulatory network was put forward. The genes associated with signal sensing, protein synthesis and degradation, carbohydrate and energy metabolism and ASA–GSH cycle for ROS scavenging as well as several key miRNAs and metabolites were crucially responsible for radish in response to Pb stress. Overall, the results of this study would be beneficial for further dissecting molecular mechanisms underlying other plant responses to HM stresses, and facilitate genetically effective management of HM contamination in root vegetable crops.

## Author contributions

YW designed the experiments and drafted the manuscript. LX, MT and WZ participated in the design of the study and performed the data analysis. MT, RW and HJ planted radish seedlings and collected samples. LX and WC reviewed and edited the manuscript. LL conceived of the study, and participated in its design and coordination and helped to draft the manuscript. All authors read and approved the final manuscript.

### Conflict of interest statement

The authors declare that the research was conducted in the absence of any commercial or financial relationships that could be construed as a potential conflict of interest.

## Supplementary material

Table S1 **The DAPS involved in radish root response under Pb500 exposure**.

Table S2 **The DAPS in radish root response under Pb1000 exposure**.

Table S3 **KEGG pathways of the DAPS involved in radish root response under Pb500 and Pb 1000 exposure**.

Table S4 **The quantitative proteins detected in the transcriptomic analysis of radish under Pb exposure**.

Table S5 **The correlated DAPS and DEGs involved in Pb stress response of radish**.

Table S6 **Association analysis of miRNAs, genes, metabolites and proteins responsive to Pb stress in radish root**.
